# The Combination of Laparoscopic and Robotic Surgery: First Experience with the Dexter Robotic System™ in Visceral Surgery

**DOI:** 10.3390/life14070874

**Published:** 2024-07-12

**Authors:** Pernilla Virginia Conrad, Anne-Sophie Mehdorn, Ibrahim Alkatout, Thomas Becker, Jan Henrik Beckmann, Julius Pochhammer

**Affiliations:** 1 Department of General, Visceral, Thoracic, Transplantation and Pediatric Surgery, University Medical Center Schleswig-Holstein, Campus Kiel, 24105 Kiel, Germany; 2 Clinic for Gynecology and Obstetrics, University Medical Center Schleswig-Holstein, Campus Kiel, 24105 Kiel, Germany

**Keywords:** robotic-assisted surgery, visceral surgery, minimally invasive surgery, Dexter robotic system, laparoscopy

## Abstract

Introduction: For over two decades, abdominal surgical procedures have been safely performed robotically. After the first patent expiration, alternative robotic systems entered the market. The Dexter Robotic System™ is a small-format, modular, and robotic platform consisting of a surgeon’s console, two patient carts with instrument arms, and one endoscope arm. We report our initial experiences with Dexter since its installation at our visceral surgery department. Methods: The system and surgical setup are described. Demographic and perioperative data of all operated patients as well as the system docking times were analyzed. Results: From 56 procedures performed with Dexter, the most common ones included cholecystectomy (*n* = 15), inguinal hernia repair (TAPP; unilateral *n* = 15; bilateral *n* = 3), and right oncologic hemicolectomy (*n* = 15). The median docking time was 6 min (2–16 min) and was reduced to 4 min in the last tertile of procedures performed. Conclusions: In our experience, Dexter can be implemented without any major challenges, and visceral surgical procedures of simple to medium complexity can be performed safely. The simplicity and accessibility of the system along with the ease of switching between robotics and laparoscopy could be particularly suitable for beginners in robotic surgery

## 1. Introduction

In modern times, we are fortunate to witness a transformation in surgery. We have come a long way since the concept of open surgery. The second half of the twentieth century, in particular, was characterized by enormous technical and surgical advances [1,2]. More recently, the advent of robotic systems has helped to improve technical precision and ease of use and reduce complications, resulting in the number of robotic systems in Germany increasing from 100 in 2018 to over 2000 in 2022 [3,4,5,6]. While the use of such systems in visceral surgery has risen to 15.1% in parts of the USA, the rate in Germany was around 1% in 2020, according to Diagnosis-Related Group (DRG) statistics [5,7]. Robotic colorectal surgery is now performed worldwide, with the highest numbers of procedures in Asia and Europe performed in China and Denmark, respectively. Robotic colorectal surgery is less common in Australia and Africa with less than 100 procedures performed [8].

Robotic systems are being adopted by the surgical community in an unprecedented way and offer a transformation of minimally invasive surgery towards more complex procedures. Numerous randomized studies have demonstrated the various advantages of robotic assistance over both open and laparoscopic surgery [9,10,11,12]. The seven degrees of freedom of movement provided by robotic instrument arms enable the surgeon to regain the dexterity of their hands that was lost during laparoscopy (LAP). This makes it possible to safely perform critical surgical steps in complex procedures that require fine dissection. The robotic system also compensates for any tremor of the surgeon and enables an optimal view thanks to the high-resolution 3D optics. The camera and robotic arms can be permanently adjusted and are therefore not dependent on an assistant. In addition, the robotic systems enable an ergonomic posture and prevent muscular fatigue and unphysiological posture of the surgeon, which are often assumed in LAP [13]. However, the currently most widely used robotic system, the DaVinci^®^ (Intuitive Surgical, Inc., Sunnyvale, CA, USA), requires a lot of space, which means that it cannot be used in every operating room (OR). OR reconstruction is often not possible, which is one reason that a clinic cannot offer robot-assisted surgery (RAS) [14,15].

With the recent expiry of patents of the first robotic systems, new surgical robotic systems are entering the market. In addition to having a new competitive situation with lower costs, these systems address the disadvantages of the DaVinci^®^ platforms and solve them in their own manner [16].

In addition to high operating costs as a disadvantage, the literature often reports longer operating times for RAS when compared to LAP, which are partly caused by the long docking times of the robot. Another disadvantage of current systems is the loss of tactile sensation [17,18].

The DaVinci closed console makes it difficult to communicate with the surgical team and the assistant. Many questions arise, particularly during the training of assistants or during RAS implementation, and clear communication is a prerequisite for safe patient care. Furthermore, the surgeon console is not sterile; therefore, no direct assistance can be provided should the assistant surgeon require it [19,20]. 

Currently authorized in Europe are at least five different robotic systems for soft tissue surgery [16]. One of these is the Dexter Robotic System™ (Distalmotion SA, Epalinges, Switzerland) [14,21,22]. This relatively new robotic system has a compact and modular design with an open, sterile console, and enables a quick change from RAS to LAP without the need for docking or undocking [14].

Our hospital has extensive experience in robotic surgery with over 5700 robotic procedures since 2013, 2750 of which were abdominal surgery. We were able to introduce the Dexter system and test its usability in the visceral surgery spectrum. In addition to an overview of the patient data and perioperative results, we report on our experiences of introducing a new robotic system into an experienced DaVinci center.

## 2. Methods

### 2.1. The Dexter System

The Dexter system is an open and modular robotic system and consists of an open, ergonomic surgeon’s console, two movable instrument modules with robotic instrument arms, and a movable endoscope module that carries the endoscope arm [14]. The surgeon’s console includes the endoscope and clutch pedals, which allow the surgeon to easily control the instruments and adjust the field of view. The instrument arms on the two modules operate a series of instruments with 7 degrees of freedom and 75 degrees of angulation. The currently available robotic instruments include monopolar scissors, a monopolar hook, a bipolar Maryland dissector, a bipolar Johann grasper, and a needle holder.

The modules can be easily transported from one OR to another and stored in a space-saving manner. The compact and modular design leaves ample working space for the table assistant and the surgical assistant around the patient during the operation.

The Dexter system is an open platform: it integrates the LAP video system, the 3D/fluorescence imaging system, and energy devices already available at the hospital, so that no new systems need to be purchased. Standard laparoscopic trocars can be used with the Dexter system. The endoscope arm can anchor any 3D endoscope, can be attached to the module or alternatively to the rail of the operating table, and is controlled from the surgeon’s console. In our case, the optics system from Karl Storz (TipCam^®^1 S 3D Lap 30°, Image1 S™ D3-Link, Image1 S™ Connect II, Karl Storz SE & Co. KG, Tuttlingen, Germany) was used. 

**Figure 1:**
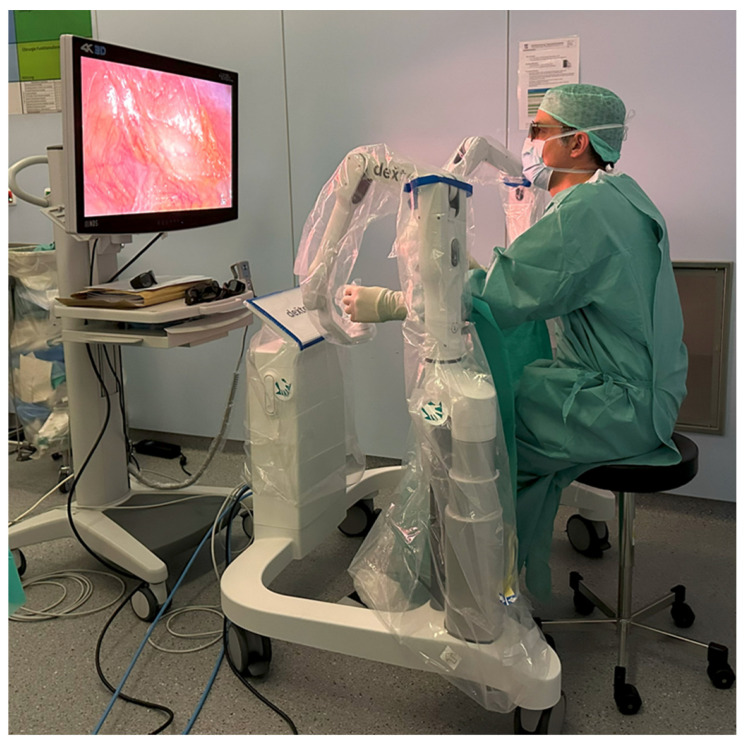
Open, sterile surgeon’s console.

**Figure 2:**
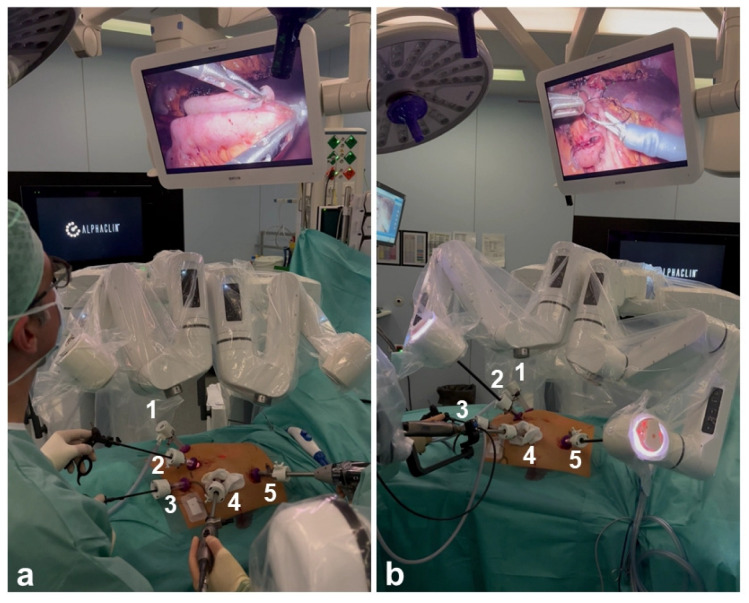
The Dexter is shown in LAP and RAS mode during the same procedure. 1–5 indicate the trocars. (**a**) Dexter arms are folded in LAP mode; (**b**) Dexter arms are connected in RAS mode.

The Dexter system combines LAP with RAS and enables rapid switching between these two approaches. The surgeon’s console is covered with sterile disposable covers (Figure 1). This allows the console surgeon to remain sterile throughout the procedure, enabling a quick change from RAS to LAP in an average of 20 s. The robotic arms do not have to be undocked to switch between LAP and RAS, but can be folded into a compact position (LAP mode) at the touch of a button, leaving the surgeon enough space for patient access (Figure 2). If it becomes apparent, intraoperatively, that a more extensive surgical treatment is necessary (e.g., carcinoma of the colonic flexures), the mobilization can be performed laparoscopically and the operation can then be completed robotically. The sterile handles of the console are reusable and can be reprocessed on site in accordance with the applicable regulations.

### 2.2. Surgical Team

All procedures in this study were performed by two specialist surgeons with extensive experience in LAP and the Da Vinci Surgical System^®^ (Intuitive Surgical, Sunnyvale, CA, USA). Each surgeon had previously performed over 300 cases robotically. 

The study was approved by the local ethics committee (D525/22, 18 August 2022). Prior to performing initial procedures, the surgeons completed the competency-based training program provided by the manufacturer. It included an online course, extensive practice on a simulator console with various digital procedures, and a mix of dry-lab and wet-lab hands-on sessions, followed by 20 h of practice on a simulator console with various digital procedures.

After acquiring the manufacturer’s certification, the surgeons performed their first procedure. The surgical team consisted of a specialist surgeon at the console, an assistant surgeon at the patient’s bedside, two OR nurses, an anesthesiologist, and a nurse anesthetist. All team members had to complete the training for the Dexter Robotic System. In addition to the surgical team, a clinical specialist from Distalmotion was on site during all procedures to assist with any potential technical difficulties with the robot. In addition, there was a regular exchange between the console surgeons at the various centers where the Dexter was implemented. This enabled problems that arose to be discussed together and solutions to be found.

### 2.3. Patients

This study prospectively evaluated the data of patients who underwent visceral surgery with the Dexter system between October 2022 and December 2023 at the Department of General, Visceral, Thoracic, Pediatric and Transplant Surgery at the University Medical Center Schleswig-Holstein, Campus Kiel, Germany. All patients were informed about a minimally invasive surgery and the possibility of using a robotic system and gave their written consent. For the included cases, we did not limit the indications to a selected patient population. The feasibility of Dexter-assisted operations was subject to the same prerequisites and restrictions as LAP operations. All patients who had an indication for visceral surgery of medium complexity (e.g., cholecystectomy, herniotomy, or hemicolectomy) were included. Patients with indications for more complex procedures (e.g., rectal resections, esophageal resections, or gastrectomies) were excluded. Patients with extensive abdominal surgery for whom a minimally invasive procedure was not considered appropriate were also excluded.

### 2.4. Data

The data collected included the procedure performed, the operation time (skin-to-skin time), and the duration of docking, measured from the start of the first module approaching the patient until the two incision pointers used for docking Dexter were removed from the trocars and returned to the sterile surface. In addition to the demographic data, the numbers of previous abdominal operations, perioperative complications that occurred (classified according to Clavien–Dindo (C-D) scale), and readmissions within 30 days were recorded.

After each operation, the console surgeons assessed the proportion of the operation performed robotically on a visual scale of 0–100. Technical and operation-related peculiarities were also recorded. The evaluation was purely descriptive using the statistical program IBM SPSS version 28. The docking times were compared in tertiles using the Kruskal–Wallis test and the post-hoc Dunn’s test with Bonferroni correction.

## 3. Results

During the period from November 2022 to September 2023, we performed 56 operations with the Dexter system, whereby 26 (46.4%) of the operated patients were female and 30 (53.6%) were male. The median age was 63 years with a range of 28–85 years. The median BMI was 26.8 kg/m2 with a range of 17.3–44.0 kg/m^2^. Among the patients, 23 patients (41.1%) had one previous abdominal surgery, 5 patients (8.9%) had two, and 2 patients (3.6%) had three previous abdominal surgeries. Of these, four patients (7.1%) had undergone open abdominal surgery. The median American Society of Anesthesiologists (ASA) classification was 3 with a range of 1–4.

Dexter was used on an interdisciplinary basis, so our department used it two days a week. During the implementation period, we experienced various difficulties, including the malfunction of the optical system from the hospital, which meant that fewer cases could be performed robotically than expected. For the first procedures with Dexter, we concentrated on highly standardized, low-complexity visceral procedures such as cholecystectomies and inguinal hernia repairs (TAPPs). Subsequently, as we became more familiar with the system, we expanded the spectrum to include more technically demanding operations such as colon resections. The procedures performed are shown in Table 1.

**Figure 3:**
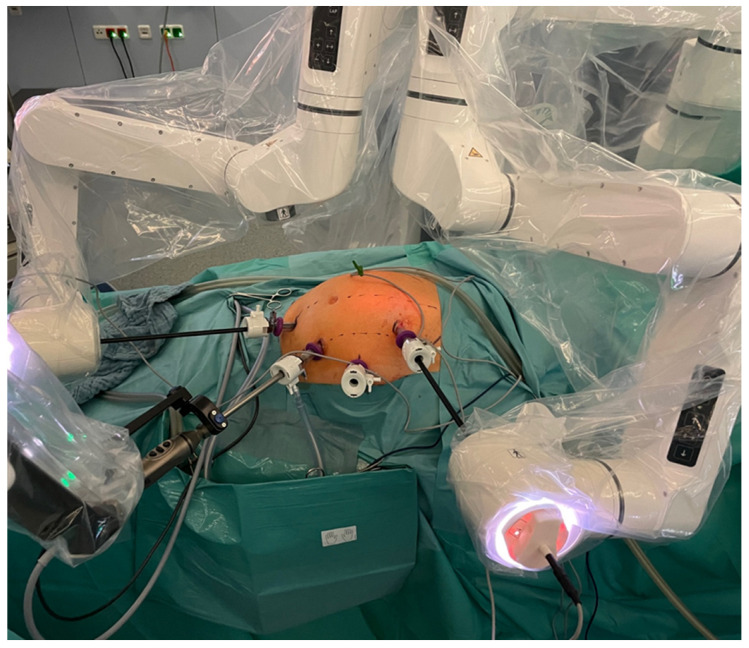
Dexter docked to the standard laparoscopy trocars with the recommended trocar position for a right hemicolectomy.

Port placement is essential for the smooth running of the procedure (Figure 3). During the learning phase, port placement had to be adjusted and procedures were sometimes completed laparoscopically. However, as Dexter integrates laparoscopic trocar placement, there was no need to add additional trocars when the decision was made to switch to LAP mode. In total, approximately 64% of the procedures were performed completely robotically. Approximately 16% were equally distributed between robotic assistance and LAP, and approximately 20% were mainly laparoscopic. Due to a suboptimal trocar position and collision of the arms, not all structures could be reached robotically in some cases, so the dissection and mobilization had to be continued laparoscopically. In addition, if there was a slight collision between the robotic arm and other structures (e.g., parts of the patient) during some movements, the system was stopped completely and had to be restarted. In some of these cases, the operation was continued laparoscopically.

**Figure 4:**
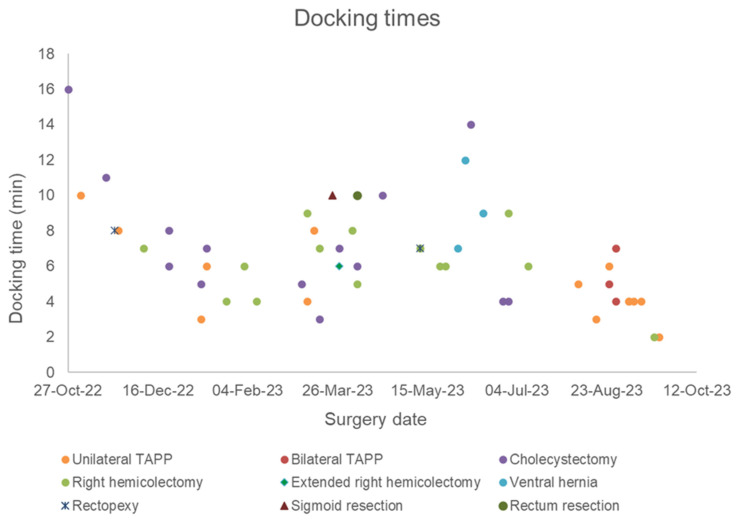
Scatter plot of the docking times by surgery date and type since the installation of the Dexter system (*n* = 53).

The first operation performed with the Dexter system was a robotic cholecystectomy in a patient with symptomatic cholecystolithiasis. This procedure was performed completely robotically, lasted 1 h 2 min, and had a docking time of 16 min. Looking at the docking times in tertiles of all procedures performed, the docking time was 6 min in the first tertile, 7 min in the second tertile, and 4 min in the last tertile, showing a significant decrease from the second tertile (*p* = 0.0005). The median docking time for all procedures was 6 min. The shortest docking time was 1 min 59 s and the longest docking time was 16 min (Figure 4).

The procedure-related times are shown in Table 1. Five operations were started with Dexter and were continued laparoscopically. The reasons for this were the robotic system (*n* = 2: a safety stop that could not be overcome due to contamination with patient secretions; 1 defective instrument), the LAP video system from the hospital (*n* = 1: a defective camera or 3D image that could not be derived), and a suboptimal trocar position (*n* = 1: central structures were not accessible during right hemicolectomy or dorsal preparation area during TAPP). One case had to be converted to an open procedure (*n* = 1). This was a planned TAPP after prostatectomy, in which the intraoperative prevesical adhesions were so extensive that it was necessary to switch to a ventral procedure (“Lichtenstein”).

Postoperative complications occurred in five cases (8.9%) (Table 2). One patient showed a decrease in hemoglobin levels of four points after a right hemicolectomy without the need for intervention (C-D 1). In another patient, a forced volume administration was necessary as a result of a pre-renal renal failure after TAPP (C-D 2). In one further case, endoscopic placement of a nutrition probe was necessary due to a pronounced intestinal atony following a right hemicolectomy (C-D 3a). One case showed an incarcerated trocar hernia after right hemicolectomy with the need for re-laparoscopy as well as anastomotic insufficiency, which necessitated a re-laparotomy for a new anastomosis (C-D 3b). The fifth complication was a postoperative bleed, which occurred after a right hemicolectomy for colon carcinoma infiltrating into the abdominal wall, requiring re-laparotomy with packing and abdominoplasty after 48 h. Iatrogenic bleeding could not be detected. Re-laparotomy with lavage for exclusion of anastomotic insufficiency in case of a paralytic ileus with aspiration pneumonia was performed (C-D 4a).

Two patients were readmitted within 30 days. In both cases, the reasons for readmission were not procedure-related. One patient suffered from a port catheter infection and exsiccosis and one patient received laxative measures for constipation.

## 4. Discussion

We reported our initial experiences with the new Dexter Robotic System from Distalmotion in visceral surgery. Dexter is a small-format, modular robotic system and can be stored in a space-saving manner and easily transported from one OR to another. This makes it particularly suitable for clinics with limited space, as the implementation of the DaVinci^®^ system requires approximately 20 m^2^ (or about 200–300 square feet) of additional space in the OR [15]. The compact and modular design led to easy storage and logistics, so that no difficulties occurred during the implementation of the Dexter.

The Dexter enables a change to laparoscopy in 20 s, and a change between the two entities is to be understood as intentional and not as a conversion. The console surgeon operates the robotic system under sterile conditions and can move to the table at any time. Other systems, however, require the robot to be completely undocked and the surgeon to be scrubbed in to enable them to work at the operating table. It is not necessary to realign (“dock”) the robotic arms when changing back to the robotic procedure. If the intraoperative findings are more extensive than expected, for example, the sterile operation of the robotic system can also be used to quickly switch to LAP and back to RAS, while with the DaVinci^®^, time-consuming and frequent re-docking is sometimes necessary [14,23].

Hahnloser et al. see this flexibility as a major advantage, as some steps in colorectal surgery can be performed faster and more efficiently laparoscopically than robotically [21].

This is also useful in the learning phase, because port placement is one of the most essential factors in RAS. This must therefore first be determined for each system and represents an important learning step when implementing new systems [23]. A suboptimal port position can result in collisions of the arms, so that not all structures can be reached for preparation. This was clearly apparent at the beginning with the Dexter. Changing to laparoscopy allows for a greater range of movement and was therefore necessary more frequently at the beginning of the learning curve in order to complete the dissection. In the meantime, Distalmotion offered cards with suggested positions for the individual procedures, which made it much easier to get started. A clinical specialist from Distalmotion supervised all procedures. The trocar positions were recorded and discussed preoperatively and procedure cards were improved with the participation of the surgeons. 

Clear, unobstructed communication in the OR is essential for training and patient safety. In our opinion, the open console and the quick change to LAP is a great advantage in the training of both console surgeons and table assistants in robotic surgery and to avoid mistakes. If the assistant surgeon has questions or uncertainties, the console surgeon can intervene and demonstrate directly, whereas with the DaVinci^®^, the console surgeon first has to undergo sterile washing again (2). The improved communication of the team can also lead to a reduction in the learning curve and operation time [14,24].

For our surgeons, who were already experienced in RAS, the familiarization with the new robotic system was quick. The free, ergonomic sitting position was perceived as positive. As described for other systems, at the beginning, there were some difficulties with the instruments [25,26]. The holding force of some instruments was also not yet fully developed at the beginning; this was later addressed and improved by Distalmotion with the new generation of instruments. At the time of writing this manuscript, ultrasonic dissectors and stackers have not yet become available for the system. Another initial challenge was the trembling of the robot arms in the peripheral areas, which was revised in the new version of Dexter. Similar to the other robotic systems, the robot arms can collide with themselves or parts of the patient during some procedures, causing the system to stop completely. These difficulties were discussed with the Distalmotion clinical expert after each operation and solutions were recently developed.

We were able to show that the standardized minor visceral surgery procedures can be performed safely with Dexter. Docking times were relatively long at the beginning, but were reduced significantly over time. When looking at docking duration over time, it is also important to consider that Dexter was used for different procedures over the course of this time, and different trocar placements required learning how to position modules differently, thus affecting the trend in the docking times. In the last tertile of performed procedures, the docking process ultimately took less than 4 min. Docking Dexter therefore became faster than the docking process with the Da Vinci, which takes an average of 10–15 min for very experienced teams after the learning curve has been overcome [27,28]. 

The operation times were also longer at the beginning compared to LAP and were able to be reduced to times comparable to LAP and Da Vinci with increasing experience. Breitenstein et al. compared robotic and laparoscopic cholecystectomies. The operation times were 55 vs. 50 min and were thus similar to our times with the Dexter [29]. The Dexter TAPP showed shorter operation times of 52 min compared to that of the Da Vinci TAPP of 71 min [30].

Compared to the initial data with the Versius Surgical System^®^ (CMR Surgical, Cambridge, UK), we showed shorter procedure times for right cholecystectomy and hemicolectomy. The complication rates were slightly lower with the Versius, with only one postoperative hemorrhage in the hemicolectomy and cholecystectomy group [31]. The complication rates for the Da Vinci hemicolectomy were reported by Hamilton et al. to be 12%, which was slightly higher in comparison to our rates [32]. We also found lower complication rates compared to those after Da Vinci TAPP. Ramser et al. published a rate of 20% [30].

Because the Dexter system is currently available with two robotic arms and one endoscope arm, we did not perform any highly complex procedures. Therefore, the range of indications for which we have chosen to use this system was limited to moderately complex procedures, but is expected to expand with further experience. These development steps are analogous to the DaVinci^®^ system, where it also took several years before highly complex procedures could be performed. However, as there is now considerably more previous experience, it can be assumed that the process will be accelerated [33].

There are also a few considerations to take into account when interpreting the results of our study. The data had to be evaluated according to the small number of cases. Our clinic has an existing robotic program, so the surgical team is used to robotic procedures. It is therefore probably easier to switch to a new system than to set up a new robotic program.

## 5. Conclusions

We report on our first experiences with the Dexter system. Standardized visceral surgical procedures are safe and easy to perform with the Dexter. The quick change to laparoscopy and the open console offer a great advantage for the training of the team and the implementation of a robotic system. Future development of the Dexter should aim to increase the range of movement and improve aiming accuracy, to then utilize the Dexter for more complex procedures.

## 6. Declaration

The robotic system was made available to the clinic to conduct an observational study, and study funds were paid to the clinic for data collection. All authors declare that there are no personal conflicts of interest.

## Figures and Tables

**Table 1:** Patient characteristics and intraoperative data according to procedures (*n* = 56).

Procedure	*n*	Age (y), Median (Range)	BMI (kg/m^2^), Median	ASA (≥3)	Operation Time, Median	Docking Time, Median
Cholecystectomy	15	54 (28–83)	27.7	6	58 min	6.5 min
TAPP, unilateral	15	60 (29–85)	26.0	8	52 min	4 min
TAPP, bilateral	3	62 (62–78)	28.1	2	100 min	5 min
Ventral hernia	3	55 (39–72)	30.1	1	139 min	9 min
Right hemicolectomy	15	73 (56–85)	26.8	13	159 min	6 min
Extended right hemicolectomy	1	78	22.6	1	222 min	6 min
Sigmoid resection	1	54	24.4	1	101 min	10 min
Rectum resection	1	39	35.1	1	123 min	10 min
Rectopexy	2	71 (64–78)	25.5	1	150 min	7.5 min

**Table 2:** Postoperative complications.

Clavien–Dindo Grade	*n*	Complication Description
1	1	Hb depletion without intervention
2	1	Scrotal hematoma, pre-renal renal failure
3a	1	Intestinal atony, Trilumen probe
3b	1	Re-laparotomy due to anastomotic insufficiency
4a	1	Re-laparotomy due to bleeding and to exclude anastomotic insufficiency, pneumonia after aspiration, ICU

## Data Availability

The original contributions presented in the study are included in the article, further inquiries can be directed to the corresponding author.
